# 离子色谱法测定呼出气和唾液中的葡萄糖

**DOI:** 10.3724/SP.J.1123.2024.06011

**Published:** 2025-03-08

**Authors:** Jianjun XU, Chaoyan LOU, Yanhong ZHUO, Yan ZHU

**Affiliations:** 1.浙江大学化学系, 浙江省微量有毒化学物健康风险评估技术研究重点实验室, 浙江 杭州 310028; 1. Department of Chemistry, Zhejiang University, Key Laboratory of Health Risk Assessment Technology for Trace Toxic Chemicals of Zhejiang Province, Hangzhou 310028, China; 2.中国计量大学质量与标准化学院, 浙江 杭州 310018; 2. College of Quality and Standardization, China Jiliang University, Hangzhou 310018, China; 3.福建医科大学附属漳州市医院放射肿瘤科, 福建 漳州 363000; 3. Department of Radiation Oncology Zhangzhou Affiliated Hospital of Fujian Medical University, Zhangzhou 363000, China

**Keywords:** 冷凝收集, 离子色谱, 呼出气, 唾液, 葡萄糖, 糖尿病, condensation collection, ion chromatography (IC), exhaled breath condensate (EBC), saliva, glucose, diabetes

## Abstract

建立了一种离子色谱测定人体呼出气和唾液中葡萄糖的方法。通过自制的呼出气冷凝装置和非刺激性方法分别收集人体呼出气和唾液,利用离子色谱对呼出气冷凝液(EBC)和唾液中的葡萄糖含量进行了检测。经过实验优化后,确定最佳冷凝温度为-14 ℃,最佳呼气流量为15 L/min。以Dionex CarboPac MA1离子色谱柱(250 mm×4 mm)为分析柱,柱温为30 ℃;以0.8 mol/L氢氧化钠溶液为流动相,泵流速设置为0.4 mL/min。在此条件下,葡萄糖在0.01~20 mg/L范围内线性关系良好,线性相关系数为0.9999,检出限为2.1 μg/L,定量限为7.0 μg/L。呼出气样品中葡萄糖含量的日内和日间精密度均≤7.5%(*n*=5),唾液样品中葡萄糖含量的日内和日间精密度均≤7.1%(*n*=5)。实验结果表明,利用该方法测定的呼出气和唾液中的葡萄糖含量与血糖水平之间均具有较好的线性关系,线性决定系数(*R*^2^)分别为0.8431和0.8204。采用该方法对6位糖尿病患者和6位健康受试者呼出气和唾液中的葡萄糖含量分别进行检测,实验结果表明,在空腹状态下,糖尿病患者和健康受试者呼出气中的葡萄糖含量差异并不大,但在口服葡萄糖1 h后,糖尿病患者呼出气中的葡萄糖含量(48.4~140.0 ng/L)比健康受试者(1.7~7.9 ng/L)高6~80倍。在空腹状态下,糖尿病患者唾液中的葡萄糖含量(87.6~158 mg/L)比健康受试者(31.6~70.9 mg/L)高1.2~5.0倍;在口服葡萄糖2 h后,糖尿病患者唾液中的葡萄糖含量(136~257 mg/L)比健康受试者(33.1~75.2 mg/L)高1.8~7.7倍。本方法采样过程简单,精密度好且对人体无副作用,可为其他人体代谢物的检测提供技术支撑。

## Abstract (en)

A novel noninasive method was developed for determining glucose levels in human exhaled breath and saliva using ion chromatography. This innovative approach involves collecting exhaled breath and saliva samples using a self-designed condensation device and non-stimulative method to ensure minimal participant discomfort. The glucose contents in both exhaled breath condensate (EBC) and saliva were analyzed using ion chromatography, which is highly sensitive and specific. The experimental conditions were optimized, including a condensation temperature of -14 ℃ and an expiratory flow of 15 L/min. A Dionex CarboPac MA1 ion chromatography column (250 mm×4 mm) was used to separate glucose, with the column temperature maintained at 30 ℃. Sodium hydroxide solution (0.8 mol/L) with a pump flow rate of 0.4 mL/min was used as the mobile phase for ion chromatography. Under these conditions, glucose exhibited a good linear relationship in the range of 0.01-20 mg/L, with a correlation coefficient of 0.9999, along with limits of detection (LOD) and quantification (LOQ) of 2.1 and 7.0 μg/L, respectively. The intra- and inter-day precisions of glucose content in exhaled breath and saliva samples of ≤7.5% (*n*=5) and ≤8.4% (*n*=5), respectively. The results reveal that the glucose levels in exhaled breath and saliva are strongly correlated with blood glucose levels. The method was validated by measuring the glucose contents of exhaled breath and saliva from six diabetic patients and six healthy subjects. Little variation in the glucose contents of the exhaled breath of the two groups was observed under fasting states. However, the exhaled breath of the diabetic patients exhibited significantly higher (by factors of 6-80) glucose contents (48.4-140.0 ng/L) than those of healthy subjects (1.7-7.9 ng/L) 1 h after glucose ingestion. Saliva samples from fasting diabetic patients contained 1.2-5.0-times more glucose contents (87.6-158 mg/L) than those of healthy subjects (31.6-70.9 mg/L). In addition, the saliva of the diabetic patients exhibited glucose contents (136-257 mg/L) that were 1.8-7.7-times higher than those of the healthy subjects (33.1-75.2 mg/L) 2 h after glucose ingestion. The developed method provides a simple, precise, and non-invasive means of detecting glucose contents in a manner that does not harm the human body; hence, it is a promising non-invasive metabolic-monitoring tool. This study opens new avenues for the development of innovative technologies for monitoring glucose and other biomarkers, which is expected to greatly enhance metabolic-study accuracy and ease, particularly in the context of managing diabetes and other metabolic disorders.

糖尿病是一种常见的慢性病,根据世界卫生组织(WHO)数据显示^[1,2]^, 2021年全球约有4.2亿糖尿病患者,预计到2030年这一数字将增加至5亿。由于目前暂无根治糖尿病的有效方法,血糖监控结合饮食管理、胰岛素注射和药物治疗是治疗糖尿病的主要措施^[3⇓-5]^。现有的血糖监测方法通常需要采集血样,这不仅会导致患者产生疼痛感,还增加了伤口感染的风险,在一定程度上限制了糖尿病患者的日常血糖监控^[6]^。因此,寻找一种非侵入性的血糖监测方法尤为重要。在各种人体易获取的体液中,气道内衬液和唾液能够与血液之间产生较快的血糖交换,因此二者可以作为非侵入式血糖监测的候选途径^[7,8]^。

唾液分析和呼出气冷凝液(exhaled breath condensate, EBC)分析均属于完全非侵入式的生物标记物采集方法。唾液通过被动流口水的方法来采集^[9]^,唾液中的主要成分是水(>99%),此外还有无机盐、蛋白质和糖类等^[10,11]^; EBC则通过对自主呼吸产生的气溶胶进行冷凝来收集,其主要成分是水(>99%),此外还有少量的气道内衬液^[12]^。与其他气道内衬液收集方法(如肺泡灌洗法和诱导痰法)不同,唾液和EBC分析不会干扰人体的正常生理过程,也不会造成任何损伤,并且可以由测试者自行完成^[13]^。然而,在水蒸气的稀释作用下,通过采集EBC所得到的葡萄糖含量通常低于10 μmol/L^[7]^,因此需要使用较为精确的分析工具来检测EBC中的葡萄糖含量。目前,研究人员已经开发了包括生物传感器^[14]^、脉冲电流^[15]^、高效液相色谱-串联质谱^[16]^和离子色谱^[17⇓-19]^等多种方法来测定EBC中的葡萄糖含量。其中,离子色谱具有准确度高、检出限低和分析速度快等特点,并且可同时对多个组分进行测定,是EBC中生物标志物的理想分析方法。

本研究设计了一种能够同时监控呼气流量、气体收集体积和冷凝温度的EBC收集装置,通过对冷凝温度和呼气流量等参数进行优化,建立了一套测定呼出气和唾液中葡萄糖含量的完整流程。同时,本研究重点探究了人体血糖水平与呼出气、唾液中葡萄糖含量的相关性,并利用口服葡萄糖耐量试验(oral glucose tolerance test, OGTT)揭示了糖尿病患者与健康人群呼出气和唾液中葡萄糖含量的差异。

## 1 实验部分

### 1.1 仪器、试剂与材料

Dionex ICS-3000离子色谱仪、Dionex ICS-5000 EC电化学检测器(工作电极为Au电极,参比电极为Ag/AgCl电极)(美国赛默飞世尔科技公司); Milli-Q超纯水机(美国Millipore公司); CPA 225D分析天平(德国Sartorius公司); KQ-500 DE数控超声波清洗器(昆山超声仪器有限公司); TGL-16GB高速离心机(上海安亭科学仪器厂);电热数显恒温水浴锅(上海达洛科学仪器有限公司);罗氏血糖仪及配套试纸(瑞士Roche公司); 0.22 μm聚四氟乙烯(PTFE)滤膜(东莞创鑫新材料有限公司); 10 mL离心管(无锡耐思生命科技股份有限公司);一次性吹嘴(扬州康安科技有限公司);葡萄糖、果糖、蔗糖和氢氧化钠(均为分析纯)(上海阿拉丁生化科技股份有限公司);娃哈哈纯净水(杭州娃哈哈集团有限公司)。

本研究已得到浙江大学医学院附属第二医院人体研究伦理委员会批准((2023)伦审研第(0557)号)。人体EBC和唾液收集于浙江大学校医院,所有参与者均签署了书面知情同意书。

### 1.2 实验装置

EBC采集装置为实验室定制,其内部结构详见图1。该采集装置由吹气口、与吹气口相连的单向阀和流量计、内置一次性冷凝收集管的冷阱组成。在采集过程中,可通过外部控制面板对呼气流量、冷凝温度与气体体积进行实时监测。

**图1:**
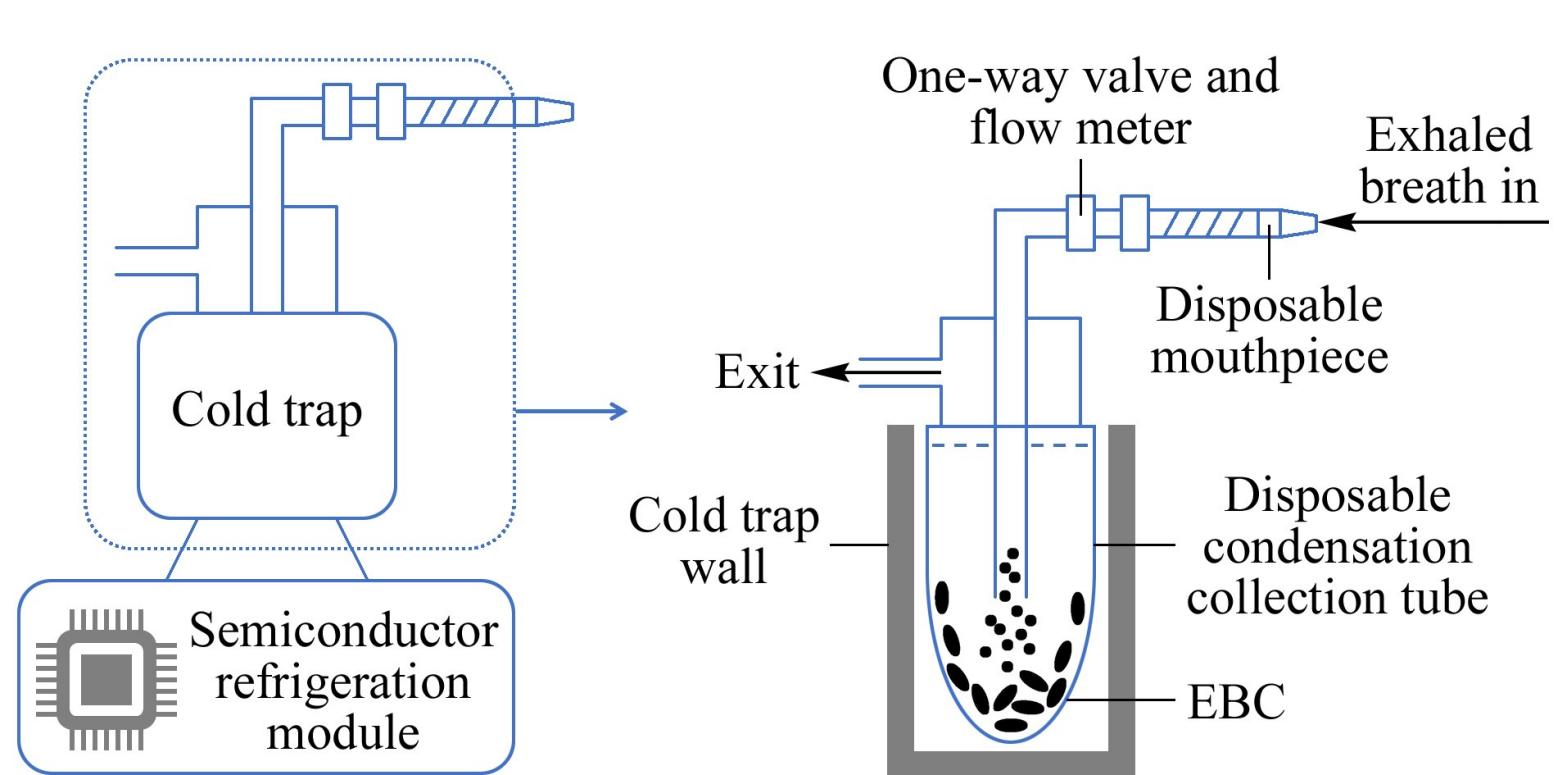
EBC采集装置示意图

### 1.3 标准溶液的配制

准确称取适量葡萄糖、果糖和蔗糖,用超纯水配制成1000 mg/L的混合标准储备液,然后再用超纯水分别逐级稀释,配制成系列质量浓度(0.01、0.05、0.1、0.5、1、2、5、10、20 mg/L)的混合标准溶液。

### 1.4 色谱条件

色谱柱:Dionex CarboPac MA1柱(250 mm×4 mm);流动相:0.8 mol/L氢氧化钠溶液,泵流速:0.4 mL/min;柱温:30 ℃;进样体积:100 μL;检测方法:脉冲安培检测法,四重脉冲波形如表1所示。

**表1:** 电位波形

Time/s	Potential/V	Integration
0	+0.1	/
0.20	+0.1	begin
0.40	+0.1	end
0.41	-2.0	/
0.42	-2.0	/
0.43	+0.6	/
0.44	-0.1	/
0.50	-0.1	/

/: null value.

### 1.5 样品的采集与分析

#### 1.5.1 EBC的采集与葡萄糖含量分析

用实验室自制的采集装置对受试者的EBC进行采集,采集前先在仪器控制面板上将冷凝温度设置为-14 ℃,呼气流量设置为15 L/min,待冷阱冷却至设定值并保持平衡时即可开始采样。受试者先用纯净水漱口,随后进行采样;在采样过程中,受试者需深吸一口气直至肺部充满气体,然后通过吹气口均匀地呼出,当达到仪器设定的采集体积时,仪器会发出提示声,此时立即停止采集;之后将一次性冷凝收集管取出,密封好后于-20 ℃冰箱中避光保存。将收集到的EBC用0.22 μm PTFE滤膜过滤,然后在1.4节条件下进行分析。扣除空白背景后,根据标准曲线计算得到EBC中的葡萄糖含量,然后利用公式(1)计算得出人体呼出气中的葡萄糖含量。


(1) $$C=\frac{C_{1}V_{1}}{V}$$

其中*C*为人体呼出气中的葡萄糖含量(ng/L), *C*_1_为EBC中的葡萄糖含量(mg/L), *V*_1_为EBC的总体积(L), *V*为呼出气的总体积(L)。

#### 1.5.2 唾液的采集与葡萄糖含量分析

受试者首先需刷牙并使用纯净水漱口30 s,然后就坐于舒适放松的位置,头部略微向前倾斜,使唾液积聚在口腔底部;弃去最初的2~3 mL唾液,然后将口腔中累积的唾液排到离心管中,直至收集到约3~4 mL唾液为止;随后将收集好的唾液样品密封、避光保存于-20 ℃冰箱中。分析前,先将唾液样品取出,放置至室温,随后将其置于95 ℃的油浴中加热35 min,以去除其中的蛋白质等杂质,然后将唾液样品自然冷却至室温,在6000 r/min下离心10 min;取出上清液,用0.22 μm PTFE滤膜过滤,滤液经超纯水稀释10倍后在1.4节色谱条件下进行分析。扣除空白背景后,根据标准曲线计算得到唾液中的葡萄糖含量。值得注意的是,在EBC和唾液的测定过程中,葡萄糖的总含量包括由果糖和蔗糖分别转化而来的葡萄糖含量。

## 2 结果与讨论

### 2.1 冷凝温度和呼气流量的优化

在健康受试者处于稳定状态且呼气流量保持恒定的情况下,将冷凝温度依次设置为-6、-8、-10、-12、-14、-16、-18、-20 ℃,在这些温度下分别进行EBC的采集与分析,每个样品平行测定3次,并利用公式(1)计算呼出气中的葡萄糖含量。在呼吸过程中,气道黏膜液以微小液滴的形式伴随挥发性物质一同被呼出。随着冷凝温度的降低,呼出气中的葡萄糖含量呈上升趋势;当冷凝温度降至-14 ℃时,冷凝过程基本完成,葡萄糖含量趋于稳定(见图2a)。因此,确定最佳冷凝温度为-14 ℃。

**图2:**
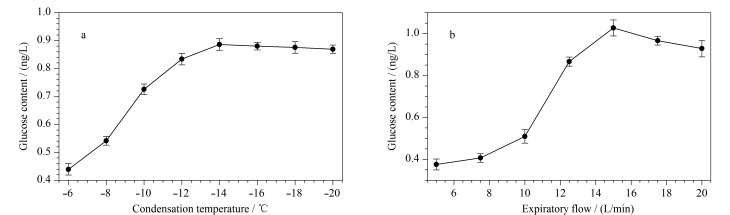
不同(a)冷凝温度和(b)呼气流量对呼出气中葡萄糖含量测定结果的影响(*n*=3)

在健康受试者处于稳定状态时,将冷凝温度设置为-14 ℃,在呼气流量分别为5、7.5、10、12.5、15、17.5、20 L/min的条件下进行EBC的采集与分析,每个样品平行测定3次,并利用公式(1)计算呼出气中的葡萄糖含量。如图2b所示,随着呼气流量的增加,呼出气中的葡萄糖含量先升高、后降低,当呼气流量为15 L/min时,所测得的葡萄糖含量最大。如果呼气流量过高,呼出气中的成分来不及冷凝就会被排出;如果呼气流量过低,部分液滴会在橡胶管道中发生冷凝,导致无法被有效收集。因此,确定最佳呼气流量为15 L/min。

### 2.2 方法学验证

#### 2.2.1 线性范围、检出限和定量限

配制质量浓度分别为0.01、0.05、0.1、0.5、1、2、5、10、20 mg/L的葡萄糖、果糖和蔗糖混合标准溶液,经0.22 μm PTFE滤膜过滤后,在1.4节色谱条件下进行分析。以质量浓度为2 mg/L的混合标准溶液为例,相应的色谱图如图3所示。扣除空白样品背景后,以峰面积为纵坐标(*y*)、质量浓度为横坐标(*x*, mg/L)进行线性拟合。分别以3倍信噪比和10倍信噪比确定检出限(LOD)和定量限(LOQ),结果见表2。实验结果表明,葡萄糖在0.01~20 mg/L范围内具有良好的线性关系,果糖和蔗糖在0.05~20 mg/L范围内具有良好的线性关系,相关系数(*r*)均≥0.9995;葡萄糖、果糖和蔗糖的LOD分别为2.1、11.3和8.7 μg/L。

**图3:**
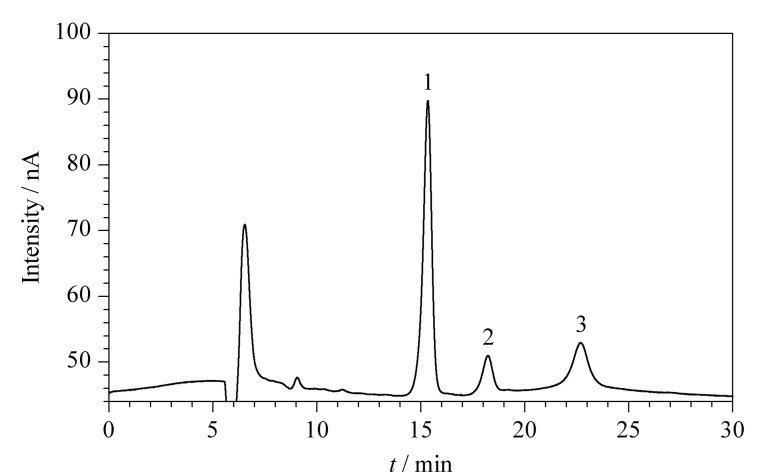
葡萄糖、蔗糖和果糖混合标准溶液(2 mg/L)的色谱图

**表2:** 葡萄糖、果糖和蔗糖的线性方程、线性范围、相关系数(*r*)、检出限和定量限

Analyte	Linear equation	Linear range/(mg/L)	r	LOD/(μg/L)	LOQ/(μg/L)
Glucose	y=16.587x-0.5084	0.01-20	0.9999	2.1	7.0
Fructose	y=2.0131x-0.3375	0.05-20	0.9995	11.3	37.7
Sucrose	y=7.1551x-1.0988	0.05-20	0.9998	8.7	28.9

*y*: peak area; *x*: mass concentration, mg/L.

#### 2.2.2 日内和日间精密度

在空腹且稳定的状态下,对健康受试者和糖尿病患者分别进行EBC的采集与分析。按照1.5.1节方法采集EBC,并于1 d内测定5次,计算得到健康受试者和糖尿病患者呼出气中葡萄糖含量的日内精密度;于一周内连续测定5 d,计算得到健康受试者和糖尿病患者呼出气中葡萄糖含量的日间精密度。实验结果表明,健康受试者呼出气中葡萄糖含量的日内和日间精密度分别为5.9%和7.5%;糖尿病患者呼出气中葡萄糖含量的日内和日间精密度分别为2.7%和3.4%。

在空腹且稳定的状态下,对健康受试者和糖尿病患者分别进行唾液的采集与分析。按照1.5.2节方法采集唾液,并在1 d内测定5次,计算得到健康受试者和糖尿病患者EBC中葡萄糖含量的日内精密度;于一周内连续测定5 d,计算得到健康受试者和糖尿病患者EBC中葡萄糖含量的日间精密度。结果表明,健康受试者唾液中葡萄糖含量的日内和日间精密度分别为3.9%和7.1%;糖尿病患者唾液中葡萄糖含量的日内和日间精密度分别为3.3%和4.4%。以上实验结果表明,本方法在分析人体呼出气和唾液中的葡萄糖含量时展现出了良好的精密度。

### 2.3 呼出气和唾液中的葡萄糖含量与血糖水平之间的线性关系

对处于空腹状态下的6位糖尿病患者(A~F)和6位健康受试者(G~L)进行OGTT。12位受试者中,A、B、C、G、H、I为成年男性,D、E、F、J、K、L为成年女性。首先将75 g葡萄糖溶于300 mL纯净水中,要求所有健康受试者和糖尿病患者于5 min内喝完。在空腹状态和口服葡萄糖后的0.5、1、2 h分别进行EBC的采集与分析,在空腹状态和口服葡萄糖后的2 h进行唾液的采集与分析,同时在各个时间点用罗氏血糖仪测试每一位健康受试者和糖尿病患者的血糖水平。计算人体呼出气和唾液样本中的葡萄糖含量,获得葡萄糖含量与血糖水平一一对应的数据,将这些数据作散点图,并进行线性拟合。结果如图4a和4b所示,呼出气和唾液中的葡萄糖含量与血糖水平之间的线性拟合程度较好,线性决定系数(*R*^2^)分别为0.8431和0.8204。

**图4:**
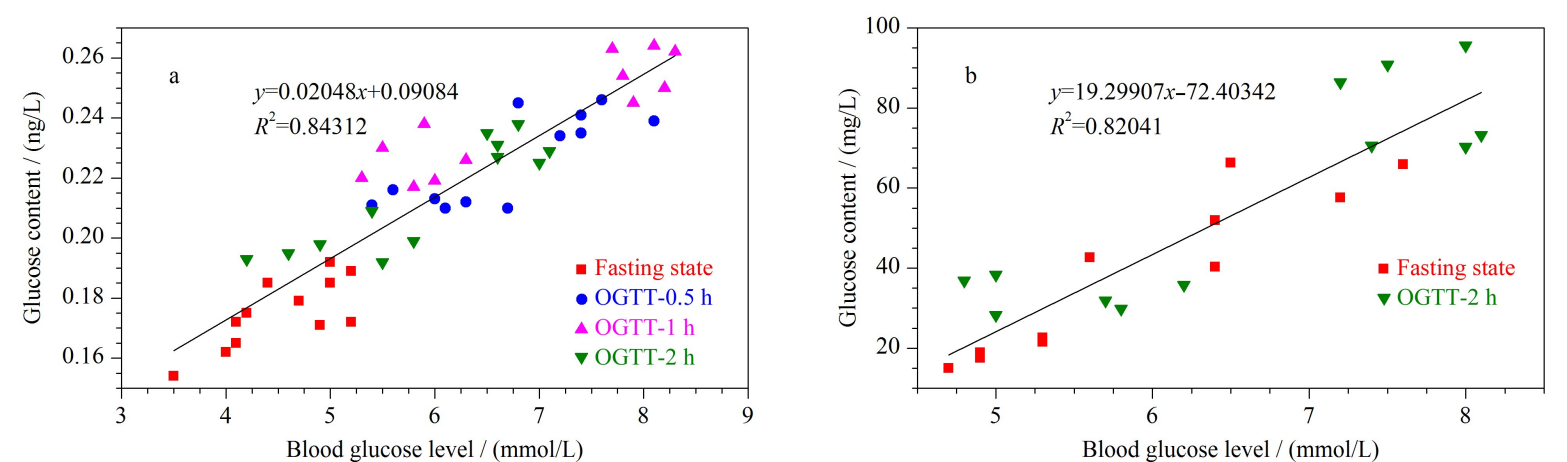
(a)呼出气及(b)唾液中的葡萄糖含量与血糖水平之间的线性关系

### 2.4 糖尿病患者和健康受试者在OGTT期间的葡萄糖含量变化

对6位糖尿病患者和6位健康受试者在空腹状态及口服葡萄糖后的0.5、1、2、3 h分别进行EBC的采集与分析,在空腹状态和口服葡萄糖后的2 h分别进行唾液的采集与分析,每个样品平行测定5次。分别计算呼出气和唾液样本中的葡萄糖含量及相对标准偏差(RSD)。由表3可知,在空腹状态下,糖尿病患者和健康受试者呼出气中的葡萄糖含量分别为0.3~1.2 ng/L和0.3~0.6 ng/L;口服葡萄糖0.5 h后,糖尿病患者和健康受试者呼出气中的葡萄糖含量分别为15.1~74.6 ng/L和0.7~1.8 ng/L;口服葡萄糖1 h后,糖尿病患者和健康受试者呼出气中的葡萄糖含量分别为48.4~140.0 ng/L和1.7~7.9 ng/L;口服葡萄糖2 h后,糖尿病患者和健康受试者呼出气中的葡萄糖含量分别为3.1~26.2 ng/L和0.4~0.9 ng/L;口服葡萄糖3 h后,糖尿病患者和健康受试者呼出气中的葡萄糖含量分别为0.4~1.2 ng/L和0.4~0.5 ng/L。由表4可知,在空腹状态下,糖尿病患者和健康受试者唾液中的葡萄糖含量分别为87.6~158 mg/L和31.6~70.9 mg/L;口服葡萄糖2 h后,糖尿病患者和健康受试者唾液中的葡萄糖含量分别为136~257 mg/L和33.1~75.2 mg/L。

**表3:** 糖尿病患者和健康受试者呼出气中的葡萄糖含量及相对标准偏差(*n*=5)

No.	Fasting state			OGTT-0.5 h			OGTT-1 h			OGTT-2 h			OGTT-3 h		
	C/(ng/L)	RSD/%		C/(ng/L)	RSD/%		C/(ng/L)	RSD/%		C/(ng/L)	RSD/%		C/(ng/L)		RSD/%
A	1.2	3.4		26.0	1.3		57.3	0.9		4.5	7.3		1.2		8.4
B	0.9	6.2		57.5	0.8		140.0	1.2		3.1	1.5		1.0		3.2
C	0.3	8.2		36.1	2.2		50.3	1.5		12.7	2.6		0.4		7.3
D	0.8	4.3		46.2	2.4		98.1	1.3		26.2	3.2		0.9		9.9
E	0.5	7.4		15.1	5.6		48.4	5.1		4.7	8.4		0.5		7.0
F	0.6	6.0		74.6	1.7		123.0	4.7		10.2	3.7		0.7		5.8
G	0.4	7.8		0.9	7.4		3.6	8.8		0.4	7.1		0.5		8.4
H	0.6	7.0		1.8	5.2		5.8	8.1		0.9	6.8		0.5		7.5
I	0.4	5.9		1.6	3.6		7.9	6.3		0.8	4.5		0.4		8.3
J	0.4	7.0		1.2	5.0		1.7	6.1		0.7	5.9		0.4		7.4
K	0.4	8.4		0.7	6.4		3.4	3.8		0.7	7.3		0.4		7.1
L	0.3	7.1		1.5	3.4		3.4	5.4		0.7	5.1		0.4		3.5

*C*: glucose content in exhaled breath; A-F: diabetic patients; G-L: healthy subjects.

**表4:** 糖尿病患者和健康受试者唾液中的葡萄糖含量及相对标准偏差(*n*=5)

No.	Fasting state			OGTT-2 h	
	C_2_/(mg/L)	RSD/%		C_2_/(mg/L)	RSD/%
A	158.0	7.9		186.0	5.8
B	124.0	5.0		257.0	3.5
C	87.6	7.0		186.0	5.0
D	126.0	6.3		176.0	9.4
E	109.0	4.2		154.0	4.0
F	93.6	3.3		136.0	3.3
G	65.2	4.9		63.3	5.6
H	50.3	4.6		52.3	5.9
I	43.8	8.2		49.7	7.8
J	70.9	4.5		75.2	6.0
K	52.8	7.0		57.2	4.8
L	31.6	7.7		33.1	8.7

*C*_2_: glucose content in saliva.

由表3可知,糖尿病患者和健康受试者呼出气中的葡萄糖含量均呈现先升高、后降低的趋势。分析其原因,人体在短时间内摄入大量葡萄糖会导致呼出气中的葡萄糖含量升高,之后在人体代谢及胰岛素的调节作用下,葡萄糖被转移至肝脏进行分解和转换,从而使呼出气中的葡萄糖含量呈现下降趋势。实验结果表明,在空腹状态下,糖尿病患者和健康受试者呼出气中的葡萄糖含量差异并不大,但在口服葡萄糖1 h后,糖尿病患者呼出气中的葡萄糖含量比健康受试者高6~80倍。由表4可知,在空腹状态下,糖尿病患者唾液中的葡萄糖含量比健康受试者高1.2~5.0倍;在口服葡萄糖2 h后,糖尿病患者唾液中的葡萄糖含量比健康受试者高1.8~7.7倍。与健康受试者相比,糖尿病患者的胰岛功能相对较弱,其对于葡萄糖的调控能力较差,因此两类人群呼出气和唾液中的葡萄糖含量存在差异。

## 3 结论

本文建立了一种非侵入式冷凝收集-离子色谱测定人体呼出气和唾液中葡萄糖含量的分析方法。通过自制的呼出气冷凝装置和非刺激性方法分别对人体呼出气和唾液进行收集,利用离子色谱进行葡萄糖含量的检测。实验结果表明,在OGTT过程中,糖尿病患者和健康受试者呼出气及唾液中的葡萄糖含量存在差异。该方法的采样过程简单,不会使受试者产生不适或面临风险,可为其他人体代谢物的检测提供技术支撑。
